# Association of Adiponectin With Cancer and All-Cause Mortality in a Japanese Community-Dwelling Elderly Cohort: A Case-Cohort Study

**DOI:** 10.2188/jea.JE20170087

**Published:** 2018-08-05

**Authors:** Reiji Kojima, Shigekazu Ukawa, Wenjing Zhao, Koji Suzuki, Hiroya Yamada, Kazuyo Tsushita, Takashi Kawamura, Satoe Okabayashi, Kenji Wakai, Hisashi Noma, Masahiko Ando, Akiko Tamakoshi

**Affiliations:** 1 Department of Public Health, Hokkaido University Graduate School of Medicine, Hokkaido, Japan; 2 Department of Pediatrics, Japan Ground Self Defense Forces Sapporo Hospital, Hokkaido, Japan; 3 Faculty of Medical Technology, Fujita Health University School of Health Sciences, Aichi, Japan; 4 Department of Hygiene, Fujita Health University School of Medicine, Aichi, Japan; 5 Comprehensive Health Science Center, Aichi Health Promotion Foundation, Aichi, Japan; 6 Kyoto University Health Service, Kyoto, Japan; 7 Department of Preventive Medicine, Nagoya University Graduate School of Medicine, Aichi, Japan; 8 Department of Data Science, The Institute of Statistical Mathematics, Tokyo, Japan; 9 Center for Advanced Medicine and Clinical Research, Hospital, Nagoya University, Aichi, Japan

**Keywords:** adiponectin, cancer mortality, community-dwelling elderly, case-cohort study

## Abstract

**Background:**

Most studies of plasma adiponectin (APN) and mortality among community-dwelling elderly focus on cardiovascular disease, but data on the relationship between plasma APN and cancer mortality is exiguous. We investigated whether APN is associated with cancer mortality in community-dwelling elderly people.

**Methods:**

We conducted a case-cohort study within the New Integrated Suburban Seniority Investigation (NISSIN) Project using a randomly drawn sub-cohort of 697 subjects (351 men and 346 women; mean age 64.5 [standard deviation, 0.5] years) among whom we compared cases of all-cause death (*n* = 269) and cancer death (*n* = 149) during a mean follow-up duration of 10.8 (standard deviation, 3.7) years. Associations between APN and mortality were assessed using weighted Cox regression analyses.

**Results:**

We observed significant positive associations between the APN concentration and cancer death in the first and third APN tertiles compared with the second APN tertile (hazard ratio [HR]_T1 vs T2_, 1.67; 95% confidence interval [CI], 1.00–2.79 and HR_T3 vs T2_, 2.10; 95% CI, 1.30–3.40). Further adjustment for possible confounders attenuated the association (HR_T1 vs T2_, 1.63; 95% CI, 0.93–2.84 and HR_T3 vs T2_, 2.10; 95% CI, 1.26–3.50). A similar but weaker association was seen for all-cause mortality (multivariate HR_T1 vs T2_, 1.45; 95% CI, 0.95–2.21 and HR_T3 vs T2_, 1.51; 95% CI, 1.01–2.25).

**Conclusion:**

Plasma APN and cancer mortality have a significant relationship among community-dwelling elderly people, which warrants further study.

## INTRODUCTION

Adiponectin (APN) is an adipokine secreted from visceral adipocytes^1^ and reportedly has insulin-sensitizing, anti-arteriosclerotic, anti-inflammatory, anti-proliferative, and anti-carcinogenic properties.^2^^–^^5^ Cross-sectional studies have also shown inverse relationships with central obesity, type 2 diabetes, and metabolic syndrome,^6^^–^^8^ although results vary regarding the onset of heart disease.^9^^–^^11^ However, longitudinal studies have shown that higher APN concentrations are associated with total and cardiovascular disease (CVD) mortality^12^^–^^20^; this is termed the “adiponectin paradox,”^21^ and its mechanism has not been sufficiently elucidated.

APN has also been considered a “protective adipokine” for cancer based on findings from cross-sectional studies. In addition, a systematic review showed an association between the APN concentration and lower risks of various cancers in case-control studies.^5^ However, another study revealed a positive association between APN and cancer death,^22^ which implies another “APN paradox” for cancer. Few reports have studied the association between APN and cancer mortality in community settings. In a nested case-control study, Matsumoto et al^23^ reported a positive but statistically marginal association between APN and cancer mortality in the low concentration range.

Cancer is a major cause of death among elderly people, especially in Japan. Vital statistics showed that, among Japanese people in their seventies, cancer accounted for about 40% of deaths, followed by heart disease (about 13%) and cerebrovascular disease (about 10%).^24^ Therefore, cancer is a more logical focus than CVD when considering the relationship between APN and mortality in the general Japanese elderly population.

In this study, we evaluated the association of cancer and all-cause mortality with adiponectin among elderly Japanese people in a community setting.

## MATERIALS AND METHODS

### Participants and study design

We conducted a case-cohort study using the New Integrated Suburban Seniority Investigation (NISSIN) Project, which is an age-specific prospective cohort study.^25^ From 1996 through 2005, all residents living in Nisshin city, Japan, aged 64 years as of the first day of each year, were invited for comprehensive health check-ups and recruited for a cohort study every year. In total, 3,073 participants (1,548 men and 1,525 women) were recruited. At baseline, each participant underwent a comprehensive health check-up, completed a detailed self-administered questionnaire, and provided an overnight fasting blood sample. Concurrently, plasma from each consenting participant was stored in freezers at −80°C. The self-administered questionnaires included information on comorbidities and demographic and lifestyle factors. All participants provided informed consent; oral consent was obtained using an opt-out approach from 1996 through 2001, and written consent was obtained using an opt-in approach from 2002 through 2005.^25^ The study was approved by the ethics committees of Nagoya University Graduate School of Medicine, the National Center for Geriatrics and Gerontology of Japan, the Aichi Medical University, and Hokkaido University Graduate School of Medicine.

### Case identification and sub-cohort selection

**Figure 1.**
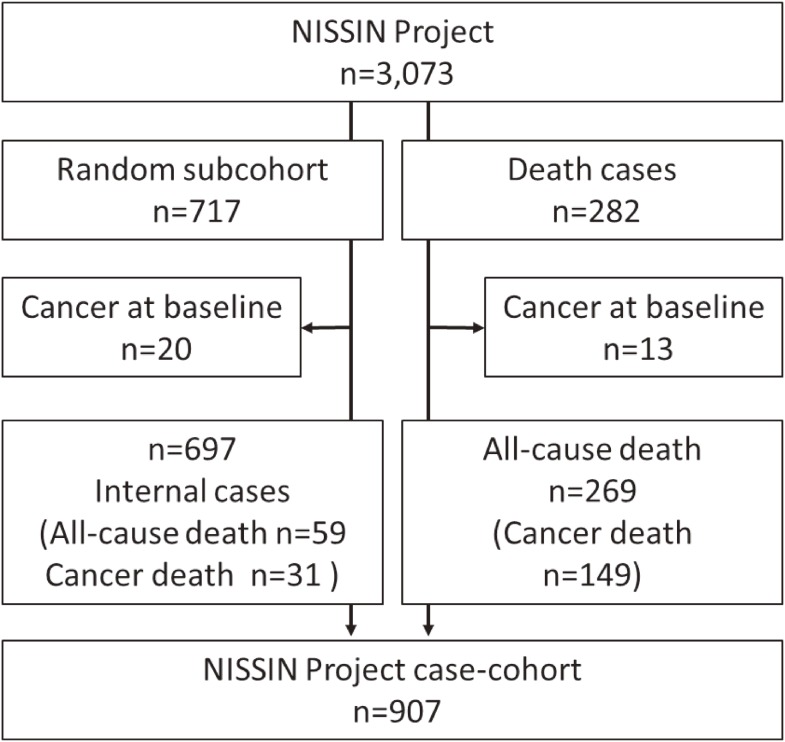
Study population schematic

From the base cohort, all cases of death were identified from complete follow-up rounds through the end of December 2012. Information regarding mortality or relocation out of the city was obtained from the city’s basic resident register. Causes of death were ascertained using the official death certificate, which was permitted by the Ministry of Health, Labour and Welfare of Japan. For the sub-cohort, we randomly selected 717 participants (about 25% of participants of each sex and registered year) (Figure 1). After excluding participants with current self-reported cancer at baseline (*n* = 20), the sub-cohort comprised 697 participants (351 men and 346 women; mean age, 64.5 [standard deviation {SD}, 0.5] years). After excluding participants with current self-reported cancer at baseline (*n* = 13) from 282 all-cause death cases, the all-cause death cases comprised 269 participants (191 men and 78 women; mean age, 64.6 [SD, 0.5] years). Of these, 59 all-cause death cases were duplicated in the sub-cohort. Based on the above data, a case-cohort data set comprising 907 participants was created (Figure 1).

### Definition of covariates

Hypertension was defined as the use of an antihypertensive medication or a blood pressure of ≥140/90 mm Hg at rest.^26^ Diabetes mellitus was defined as a previous diagnosis with ongoing treatment or a fasting blood glucose concentration of ≥126 mg/dL.^27^ Dyslipidemia was defined as a previous diagnosis with ongoing treatment, a triglyceride concentration of ≥150 mg/dL, or high-density lipoprotein cholesterol concentration of <40 mg/dL.^28^ CVD was defined as a history of angina pectoris, myocardial infarction, or stroke. The body mass index (BMI) was calculated as the measured weight in kilograms divided by the measured height in meters squared and was categorized into two groups: <25.0 and ≥25.0 kg/m^2^.

### Measurement of biomarkers

The total APN and C-reactive protein (CRP) concentrations were assayed in 2015–2016 from baseline plasma samples that had been stored at −80°C and had never been thawed. Adiponectin was measured with the Human Adiponectin ELISA, High Sensitivity Kit (Biovendor Laboratory Medicine, Brno, Czech Republic); the intra-assay and inter-assay coefficients of variation were 3.3%–4.4% and 5.8%–6.2%, respectively, according to the instruction manual. CRP was measured with CRP-Latex (II) X2 “Seiken” (Denka Seiken Co., Ltd, Tokyo, Japan); the intra-assay and inter-assay coefficients of variation were both <10%. The measurers were not informed of distinctions between the cases or sub-cohorts. The APN and CRP concentrations were adjusted by the ratios of the baseline/current measured total protein level based on the years of blood sample storage.

### Statistical analysis

Hazard ratios (HRs) and their 95% confidence intervals (CIs) for associations of APN with cancer mortality and all-cause mortality were calculated using weighted Cox regression analyses for stratified case-cohort studies.^29^^,^^30^ The APN concentrations were grouped into tertiles based on the sub-cohort only, with the middle tertile set as the reference. First, we created a crude model, adjusting for sex as needed. In the second model, we added further possible confounding factors with reference to a previous study,^23^ including smoking (“never,” “former,” “current”), drinking (“never,” “current”), BMI (<25.0 or ≥25.0 kg/m^2^), hypertension, diabetes mellitus, dyslipidemia, CVD, and CRP (log-transformed, mg/dL, continuous). Quadratic trends were tested by treating tertile medians as quantitative exposure scores. All analyses were stratified by the enrollment year. HRs were calculated using a Cox proportional-hazards model modified by Barlow weights to account for the case-cohort design. We confirmed the assumption of proportional hazards for APN using Schoenfeld residual plots, which suggested that the fitted model was satisfactory. We conducted a sensitivity analysis that excluded patients with diabetes mellitus at baseline to check for reverse causality. All quoted *P*-values are two-sided. All statistical analyses were carried out using SAS version 9.4 (SAS Institute, Cary, NC, USA).

## RESULTS

Among the 3,073 subjects, 269 died of all causes, including 149 (55.4%) of cancer and 39 (14.5%) of CVD, during the mean follow-up of 10.8 (SD, 3.7) years, after excluding subjects with current cancer at baseline (*n* = 13) (Figure 1). Of these, 59 all-cause death cases and 31 cancer death cases were duplicated in the sub-cohort (Figure 1). Cancer mortality by site among men was as follows (total, *n* = 104): lung, *n* = 35; stomach, *n* = 17; colon, *n* = 9; liver, *n* = 3; pancreas, *n* = 6; prostate, *n* = 5; esophagus, *n* = 5; gall bladder, *n* = 6; bladder, *n* = 2; lymphatic tissue, *n* = 4; hematopoietic tissue, *n* = 7; and others, *n* = 5. Among women, cancer mortality by site was as follows (total, *n* = 45): lung, *n* = 10; stomach, *n* = 4; colon, *n* = 9; liver, *n* = 3; pancreas, *n* = 5; esophagus, *n* = 2; breast, *n* = 3; uterus, *n* = 1; ovary, *n* = 1; gall bladder, *n* = 2; lymphatic tissue, *n* = 1; hematopoietic tissue, *n* = 1; and others, *n* = 3. The mean APN concentration in the sub-cohort were as follows: men, 7.5 (SD, 3.3) mg/L and women, 11.3 (SD, 5.2) mg/L; these concentrations among the death cases were as follows: men, 7.7 (SD, 3.8) mg/L and women, 11.3 (SD, 4.7) mg/L. Table 1 shows the subjects’ baseline characteristics by APN tertiles and sex. For both sexes, those in higher APN tertiles were more likely to have lower proportions of dyslipidemia. For women, those in higher APN tertiles were more likely to have a lower BMI and lower proportions of hypertension and diabetes mellitus.

**Table 1.** Baseline characteristics by sub-cohort

		Male (*n* = 351)			Female (*n* = 346)		
	
		T1 (*n* = 117)	T2 (*n* = 117)	T3 (*n* = 117)	T1 (*n* = 115)	T2 (*n* = 115)	T3 (*n* = 116)
	Adiponectin range (mg/L)	<6.0	6.0–8.1	≥8.1	<8.6	8.6–12.5	≥12.5
Marital status, *n* (%)	Married	113 (96.6)	110 (94.8)	110 (95.7)	98 (85.2)	91 (79.1)	93 (81.6)
	Divorced/Widowed/Single	4 (3.4)	5 (4.3)	5 (4.3)	17 (14.8)	24 (20.1)	21 (18.4)
Education, *n* (%)	Elementary/Junior	28 (23.9)	38 (32.8)	41 (35.3)	47 (40.1)	35 (30.4)	36 (31.3)
	High school	49 (41.9)	39 (33.6)	37 (31.9)	53 (46.1)	65 (56.5)	64 (55.7)
	College/university above	42 (35.9)	38 (32.8)	38 (32.8)	15 (13.0)	15 (13.0)	15 (13.0)
Smoking, *n* (%)	Never	21 (18.0)	20 (17.1)	24 (20.5)	107 (93.0)	103 (89.6)	103 (88.8)
	Past	58 (49.6)	64 (54.7)	56 (47.9)	6 (5.2)	7 (6.1)	3 (2.6)
	Current	38 (32.5)	33 (28.2)	37 (31.6)	2 (1.7)	5 (4.4)	10 (8.6)
Drinking, *n* (%)	Non	39 (33.3)	45 (38.5)	39 (33.3)	94 (81.7)	90 (78.3)	84 (72.4)
	Current	78 (66.7)	72 (61.5)	78 (66.7)	21 (18.3)	25 (21.7)	32 (27.6)
BMI, *n* (%)	<25 kg/m^2^	85 (72.7)	85 (72.7)	94 (80.3)	82 (71.3)	100 (87.0)	106 (91.4)
	25 kg/m^2^≤	32 (27.4)	32 (27.4)	23 (19.7)	33 (28.7)	15 (13.0)	10 (8.6)
Comorbidity, *n* (%)	Hypertension	65 (55.6)	63 (53.9)	53 (45.3)	57 (49.6)	39 (33.9)	36 (31.0)
	Diabetes mellitus	26 (22.2)	16 (13.7)	14 (12.0)	14 (12.1)	7 (6.1)	1 (0.9)
	Dyslipidemia	68 (58.1)	48 (41.0)	30 (25.6)	47 (40.9)	29 (25.2)	20 (17.2)
	CVD	10 (8.6)	17 (14.5)	9 (7.7)	4 (3.5)	5 (4.4)	8 (6.9)
CRP, mg/L	Median	0.075	0.066	0.035	0.061	0.043	0.026
	Interquartile range	0.032–0.158	0.033–0.123	0.021–0.085	0.031–0.098	0.022–0.100	0.021–0.051

BMI, body mass index; CRP, C-reactive protein; CVD, cardiovascular disease; T, tertile.

Table 2 shows the multivariate-adjusted HRs and 95% CIs for cancer mortality and all-cause mortality by APN tertile and sex. The person-years per tertile in the sub-cohort were as follows: first tertile, 2,536.9; second tertile, 2,598.4; and third tertile, 2,419.8. A significant association with a nadir at the second APN tertile was observed for both cancer mortality and all-cause mortality. There were significant positive associations between the APN concentration and cancer death in the first and third APN tertiles compared with the second APN tertile in the sex-adjusted model (HR_T1 vs T2_, 1.67; 95% CI, 1.00–2.79 and HR_T3 vs T2_, 2.10; 95% CI, 1.30–3.40). The association was slightly attenuated in the multivariable-adjusted model (HR_T1 vs T2_, 1.63; 95% CI, 0.93–2.84 and HR_T3 vs T2_, 2.10; 95% CI, 1.26–3.50). For all-cause mortality, a similar but weaker association was also observed. Significant quadratic trends were seen for both cancer mortality and all-cause mortality, even in the multivariable-adjusted model (*P* for quadratic trends: <0.01 and 0.02, respectively). Stratified analysis by sex showed similar associations in both sexes, but the associations were not statistically significant in the multivariable-adjusted model except for male cancer mortality (HR_T3 vs T2_, 2.15; 95% CI, 1.11–4.16). The result of the sensitivity analysis that excluded patients with diabetes mellitus led to similar conclusions as obtained in the main analysis.

**Table 2.** Association of total adiponectin with cancer and all-cause mortality

Mortality		T1 (low)		T2		T3 (high)		

Total population		HR	95% CI	HR	95% CI	HR	95% CI	*P*^b^
Cancer	*n*	53		34		62		
	Sex-adjusted HR	1.67	1.00–2.79	1.00	(reference)	2.10	1.30–3.40	<0.01
	Multivariate HR^a^	1.63	0.93–2.84	1.00	(reference)	2.10	1.26–3.50	<0.01
All cause	*n*	98		74		97		
	Sex-adjusted HR	1.51	1.02–2.22	1.00	(reference)	1.47	1.01–2.13	0.02
	Multivariate HR^a^	1.45	0.95–2.21	1.00	(reference)	1.51	1.01–2.25	0.02
Male								
Cancer	*n*	39		24		41		
	Crude HR	1.82	0.96–3.46	1.00	(reference)	2.07	1.12–3.83	0.02
	Multivariate HR^a^	1.81	0.89–3.69	1.00	(reference)	2.15	1.11–4.16	0.02
All cause	*n*	70		52		69		
	Crude HR	1.56	0.98–2.53	1.00	(reference)	1.49	0.93–2.38	0.05
	Multivariate HR^a^	1.53	0.90–2.62	1.00	(reference)	1.55	0.93–2.58	0.05
Female								
Cancer	*n*	14		10		21		
	Crude HR	1.42	0.59–3.42	1.00	(reference)	2.08	0.94–4.59	0.16
	Multivariate HR^a^	1.41	0.57–3.49	1.00	(reference)	1.95	0.80–4.76	0.19
All cause	*n*	28		22		28		
	Crude HR	1.37	0.73–2.59	1	(reference)	1.32	0.71–2.47	0.29
	Multivariate HR^a^	1.32	0.66–2.65	1	(reference)	1.35	0.66–2.73	0.35

CI, confidence interval; HR, hazard ratio; T, tertile.

All analyses were stratified by enrollment year. Hazard ratios were calculated by a Cox proportional hazards model modified by Barlow weights to account for the case-cohort design.

^a^Adjusted for smoking, drinking, body mass index, hypertension, diabetes mellitus, dyslipidemia, cardiovascular disease, C-reactive protein (log-transformed), and sex (for total population).

^b^For quadratic trend.

## DISCUSSION

Examination of the association between APN and cancer mortality among community-dwelling elderly people revealed a significant association between the APN concentration and cancer death in the first and third APN tertiles compared with the second APN tertile; these findings were also revealed in the total mortality figures.

Previous studies have shown that APN and cancer prognosis (especially death) are positively related among patients with cancer.^22^ Our study extended this to community-dwelling elderly people and observed a significant association between APN and cancer mortality with a nadir in the second APN tertile. Kizer et al^15^ showed a U-shaped association between APN and CVD mortality among community-dwelling elderly participants who had no CVD or heart failure/atrial fibrillation, but APN had a direct association among subjects with heart failure/atrial fibrillation. The authors explained that, within the lower APN concentration range, the higher APN concentrations conferred a lower risk of death by exerting a useful effect, but, within the higher APN concentration range, higher concentrations reflected pathological conditions and increased the risk of death.^15^ Because our study excluded patients with cancer at baseline for analysis, the association between APN and cancer mortality might be similar to these findings regarding CVD mortality. In contrast, in a nested case-control study in which the association between APN and cancer mortality was examined in a community-dwelling population, higher APN concentrations were associated with higher risk of death in the low-APN range.^23^ Thus, the association between APN and cancer death among community-dwelling elderly people is controversial. Considering that APN has been suggested to be a biomarker for cancer^5^^,^^22^ and that cancer is the leading cause of death among the elderly,^24^ further studies on the relationship between cancer mortality and APN among community-dwelling older people are necessary.

The mechanisms that drive the association between APN and cancer mortality with a nadir in the second APN tertile are uncertain. In laboratory research, APN has reportedly exhibited an anti-cancer effect through anti-proliferative action and insulin resistance.^5^ In the low concentration range, APN might exert an anti-cancer effect as the concentration increases, whereas in the high concentration range, a higher concentration of APN might indicate APN resistance that eventually leads to compensatory upregulation and an increased risk of death.^13^^,^^21^^,^^31^ A direct effect of APN on cancer prognosis might be considered,^22^ but further studies are needed.

We observed a similarly shaped association for all-cause mortality, which is consistent with previous studies showing a U-shaped association between APN and total mortality.^14^^,^^15^ In those studies, however, the rate of death from CVD was as high as 30%, compared with 14% in the present study. Cancer mortality in the present study was 55%, which, along with our CVD rate, was close to the composition of causes of death in Japanese people in their seventies.^24^ Therefore, we consider that the all-cause mortality rate reflected the relationship between APN and cancer mortality. Further analysis, stratified by sex, showed consistent, similarly shaped associations. Although significant results were partly seen among male subjects, insufficient statistical power due to few events might explain some of our results.

The strengths of our study are its focus on the leading cause of death among elderly people (ie, cancer) the examination of the association between this leading cause of death and APN in this community-dwelling age-specific cohort, and the confirmation of the association between APN and cancer. Most cohort studies that include people with a wide range of ages adjust for age using multivariable regression. However, an unadjustable age contribution could remain because age effects are not always log-linear. Our age-specific cohort allowed us to show the associations without age-derived biases.^25^

A limitation of this study is its inclusion of various cancer types among cancer deaths. In many cancers, high APN concentrations are reportedly associated with a low cancer incidence,^5^ but liver and pancreatic cancers reportedly have the opposite relationships^32^^,^^33^; this implies that carcinomas vary in their associations between APN and cancer death. However, the number of cases was small in this study, and it was not possible to examine each carcinoma. Therefore, further studies that consider carcinoma type are warranted. In this study, only the total APN concentration was examined; high-molecular-weight (HMW) APN, which is the biologically active part of APN, was not studied.^34^ However, some studies that simultaneously measured the total APN and HMW APN concentrations and examined their associations with mortality showed that the association between HMW APN and all-cause mortality was almost the same as that with total APN^12^^,^^15^; therefore, similar results would be expected for associations between HMW APN and cancer death. Conversely, APN might have a cancer-specific effect, so the association between HMW APN and cancer death needs to be further studied.

### Conclusions

We observed a significant association between cancer mortality and the level of adiponectin in community-dwelling elderly people and confirmed a similar association for all-cause mortality. Further studies are needed to elucidate the mechanism of this association.
